# Medical and Physical Intelligence

**Published:** 1825-07

**Authors:** 


### MEDICAL AND PHYSICAL INTELLIGENCE

### PATHOLOGY.

1. ***Various Morbid Alterations in New-born Infants.***—M. Bricheteau lately read, to the Academie Royale de Medicine, a report on the work of Dr. Veron, containing three cases, in which the fœtus had been affected with inflammations, similar to those which affect the adult. In one of these, a child, which only lived twelve or fifteen hours, the post-mortem examination led to the discovery of a pleurisy,—viz. effusion of purulent fluid in the thorax, formation of false membrane on the pleura, redness and injection of vessels. In the second, the infant presented traces of peritonitis; and in the third there had been inflammation of the thymous gland, with the formation of pus in the interior of that organ. M. Bricheteau added other facts to those of M. Veron: for example, he quoted instances in which children were born with small-pox; a circumstance confirmed by the experience of various other members of the Academy. M. Desormeaux gave the history of a child who was born with every symptom of an intense enteritis of long standing, and which was cured. M. Husson lately opened, at the Hôtel Dieu, the bodies of two infants,—one born dead at the seventh month, the other which only lived eight days,—both of which had tubercles, already softened, and in a state of suppuration: the former in the lungs, although the mother was in good health; the latter in the liver. MM. Dupuy and Andral mentioned having found tubercles in the fœtuses of some of the lower animals; and M. Andral, in opening the body of a woman who died of phthisis, found the supra-renal capsules of the fœtus inflamed and suppurated.—(*Archives General*, Mai.)

---

### THERAPEUTICS.

2. *Use of the Kali Hydriodinici in Cutaneous Cancer.*—Dr. Grafe, of Berlin, has published a few short remarks upon the curative power of the kali hydriodinici in cutaneous cancer. In the case of a woman whose breast was affected with this disease, and who had in vain employed other remedies, an ointment, composed of one drachm of the above preparation and two ounces of simple ointment, was applied once a-day. The wound soon assumed a healthier appearance; the callous edges disappeared, and cicatrisation soon began. The strength of the ointment was now doubled. The healing of the ulceration proceeded rapidly, and in the course of a few weeks it was quite well.

In cancerous affections of the upper lip, also, the same preparation has been employed with much advantage.—(*Journal fur Chirurgie, &c.* Grafe and Walther; band 7, stuck i.)

3. *The Use of the Rhus Toxicodendron in Palsy of the Limbs.*—A young man, twenty years of age, of a strong frame, but stupid mind, was admitted into an hospital for a rheumatic affection, which passed off. He subsequently complained of no pain; he ate and slept well. The muscular power of his hands and feet was diminished, and they were almost useless; the fæces were sometimes passed involuntarily. As this symptom was attributed to his natural stupidity, he was threatened with punishment. The power of his limbs was now so completely lost, that he was obliged to be dressed and undressed like a child. In a short time he could not even move his fingers, and was incapable of feeding himself. Various remedies were ineffectually employed; amongst others, the phosphorus in an emulsion. Two days after the use of this article, he became perfectly jaundiced, and was attacked by fever. The relater of the case was now "at his wit's end;" he knew not what to do, and therefore determined to do nothing. In a few days, the last-mentioned symptoms passed off. The appetite returned, and the palsy of the extremities alone remained. He improved so much in general appearance, that another effort was determined upon, to endeavour to afford him relief. Upon the principle of Celsus, that it is better "anceps remedium experiri quam nullum," the rhus toxicodendron was given,—a drop night and morning. In eight days, the patient could move his fingers; in a month, the arms and feet. The dose of the remedy was gradually increased to ten drops. In two months, he had the perfect use of his limbs. He was shortly quite restored to bodily health; but still remained in a state of mental imbecility.—(*Allgemeine Med. Annalen,* Jan. 1825.)

---

### SURGERY.

4. *Large Calculus passed from the Female Urethra.*—A case is related by Dr. Sommer, in the *Journal der Chirurgie und Augen-Heilkunde,* (band 7, heft i.) in which a female, of nineteen years of age, after suffering from severe pain in passing her urine, and occasional retention of it, had a catheter introduced, by which a stone was discovered obstructing the passage. The urethra was dilated by forceps; the stone was grasped by them, and withdrawn. It was found to weigh ten drachms and a half; its long diameter measured two inches and a quarter; the transverse diameter was one inch and a quarter: it was of a whitish grey colour, tolerably hard, and rough upon its superficies. The patient soon recovered.

5. *Extirpation of the Clitoris.*—An interesting, but unnecessarily detailed, account of this operation is given in Grafe and Walther's *Journal der Chirurgie und Augen-Heilkunde,* (band 7, stuck i.) The subject of it had existed in a state of complete idiotism from a very early age. Many ineffectual endeavours were made to afford relief by several celebrated German practitioners, as it was considered that the mental affection was dependent upon corporeal disease, and therefore susceptible of cure. At the age of fifteen years, the wretched creature was still living, in a complete state of mental imbecility; and it now became necessary to confine her arms in bed, to prevent her from committing incessant masturbation. As her intellectual faculties had for a short time appeared somewhat improved, it became a matter of considerable importance to put an effectual stop to the vice, which would so certainly have been the cause of her remaining in a state of mental hebetude.

After many fruitless efforts to effect this purpose by other means, the extirpation of the clitoris was had recourse to, in the month of June. The patient was watched, and her libidinous propensities gradually decreased. From this time her mental powers slowly improved. She learnt to write her letters, and was capable of receiving other instruction. She endeavoured to atone for her imperfect powers of speech, by expressive signs. She made some progress in music.

Two conclusions are drawn from this case, by the anonymous relator of it:—

1st. That idiotism is curable, if it is not dependent upon some congenital malformation.

2d. That onanism, particularly when carried to excess, will confirm a state of idiotism.

It is observed that, although the disgusting practice of masturbation undoubtedly confirmed the mental imbecility of the patient, it might still be difficult to determine the primary cause of the idiotism. Self-pollution could hardly have taken place at a very early age, and yet the state of fatuity was complete; and the latter part of the treatment consisted almost entirely in overcoming the disposition to the commission of the vice referred to.

6. ***High Operation for the Stone.***—We have heard, through the medium of a highly respected correspondent, that the above operation has recently been performed by Mr. C. HUTCHINSON, at Sheerness, with complete success; the calculus extracted being described as " a very large one."

---

### MISCELLANEOUS.

7. *Great Hospital at Munich.*—This handsome edifice was built in 1813; it is situated without the city, from which it is completely detached. It is arranged according to the plan of Dr. HOBERL, and is under the direction of the municipal authority. One half contains the men, the other the women. There are in all fifty-four wards, each having twelve beds, placed two and two. The methods of heating and ventilating the building, are the points most worthy of attention. Stoves, which traverse each story, and which are kindled outside, serve for the purpose of warming the wards. They are surrounded in each room by a covering of iron, in such a manner as to leave a certain space between the stove and the covering. In this space, pipes open, which conduct fresh air; this mixes with the heated air, and purifies it, before it becomes distributed over the ward. To procure this supply of fresh air, the following ingenious method is adopted :—The wings of the building are flanked by hexagonal towers, open to the wind from every point. If the wind be too strong, the effect can be diminished by lattices and ventilators. From these towers, or turrets, the air passes, by means of pipes, into the wards, close by the stoves; so that the patients breathe pure air, without ever being incommoded by it.

A machine, invented by M. RHEICHENBACH, conveys water to the top of the hospital, and thence distributes it to every part of the building; so that an abundant supply is always at hand, in case of fire. To every second ward there is a kind of anti-chamber, with a stove for warming water. The linen of the patients is washed in salt water, by means of a machine for the purpose, and without manual assistance. There are particular arrangements for sulphureous fumigations. The vapour of water is brought in contact with a mixture of two parts of sulphur and one part of saltpetre: the steam impregnated with this mixture fills a machine, in which the patient is seated so as to have only the head out. These vapour-baths frequently take the place of internal remedies. There are two gardens,—one for the men, the other for the women. The principal diseases are catarrhal affections, rheumatism, and gout; phthisis is also common; epidemics are rare. The average number treated at this hospital is about 3,500.—*(Nye Hygæa.)*

---

### METEOROLOGICAL JOURNAL,

By Messrs. WILLIAM HARRIS and Co. 50, Holborn, London.

***From May 20 to June 19, 1825.***

| | MOON. | Rain gauge | THERM. | | | BAROM. | | DE LUC'S HYG. | | WIND. | | ATMOSPHERIC VARIATIONS. | | |
|---|---|---|---|---|---|---|---|---|---|---|---|---|---|---|
| | | | 9 A.M. | MAX. | MIN. | 9 A.M. | 10P.M. | 9 A.M. | 10P.M. | 9 A.M. | 10P.M. | 9 A.M. | 2 P.M. | 10P.M. |
| May | | | | | | | | | | | | | | |
| 20 | | | 53 | 62 | 49 | 30.08 | 30.04 | 79 | 62 | E | ESE | Fine | Fine | Fine. |
| 21 | | | 60 | 68 | 46 | 30.00 | 29.94 | 69 | 70 | E | ESE | | | |
| 22 | | | 60 | 68 | 50 | 29.90 | 29.90 | 67 | 62 | E | S | | | |
| 23 | | | 66 | 72 | 55 | 29.81 | 29.64 | 60 | 61 | SSW | SW | | | |
| 24 | | | 65 | 68 | 53 | 29.62 | 29.55 | 63 | 73 | WSW | S | | | |
| 25 | ☽ | | 64 | 67 | 53 | 29.50 | 29.47 | 70 | 79 | WSW | SW | | | |
| 26 | | | 61 | 63 | 44 | 29.50 | 29.54 | 73 | 81 | NW | NE | Cloud. | | Rain |
| 27 | | .14 | 51 | 56 | 42 | 29.57 | 29.67 | 71 | 60 | NNE | ENE | | | Fine |
| 28 | | | 43 | 50 | 40 | 29.72 | 29.67 | 59 | 73 | W | SE | Fine | Rain | |
| 29 | | .17 | 55 | 57 | 45 | 29.73 | 29.80 | 68 | 77 | SW | NE | Cloud. | Sho'ry | Cloud. |
| 30 | | .5 | 52 | 58 | 42 | 29.75 | 29.96 | 62 | 69 | ENE | ESE | Fine | Fine | |
| 31 | ○ | | 51 | 58 | 46 | 30.14 | 30.20 | 64 | 72 | NNE | SSE | Cloud. | Cloud. | Fine |
| June | | | | | | | | | | | | | | |
| 1 | | | 56 | 65 | 52 | 30.15 | 30.09 | 58 | 80 | ESE | WSW | Fine | Fine | Cloud. |
| 2 | | | 57 | 64 | 53 | 29.93 | 30.00 | 65 | 70 | WSW | W | Cloud. | Cloud. | |
| 3 | | | 58 | 68 | 46 | 29.96 | 29.60 | 75 | 70 | NW | W | Rain | Sho'ry | Fine |
| 4 | | .11 | 56 | 58 | 47 | 29.43 | 29.20 | 76 | 72 | WSW | W | | Rain | Cloud. |
| 5 | | .18 | 54 | 58 | 42 | 29.30 | 29.68 | 63 | 71 | NW | NW | Cloud. | Cloud. | Fine |
| 6 | | | 56 | 64 | 52 | 28.80 | 29.80 | 66 | 72 | WNW | SSW | Fine | | |
| 7 | | | 60 | 68 | 55 | 29.75 | 29.76 | 65 | 68 | SW | WSW | | | |
| 8 | ☾ | | 63 | 68 | 52 | 29.79 | 29.83 | 72 | 65 | SSW | WSW | Cloud. | Fine | |
| 9 | | | 62 | 72 | 55 | 29.90 | 30.00 | 70 | 60 | W | W | | | |
| 10 | | | 62 | 72 | 60 | 30.07 | 30.09 | 58 | 65 | W | WSW | Fine | | |
| 11 | | | 62 | 73 | 57 | 30.07 | 33.01 | 67 | 65 | SW | SE | Cloud. | | |
| 12 | | | 63 | 76 | 60 | 29.99 | 29.95 | 57 | 63 | SE | ESE | Fine | | |
| 13 | | | 68 | 78 | 57 | 29.96 | 30.04 | 63 | 55 | NE | NE | | | |
| 14 | | | 66 | 75 | 53 | 30.09 | 30.16 | 64 | 60 | NE | E | | | |
| 15 | | | 63 | 72 | 49 | 30.13 | 30.08 | 61 | 56 | NE | E | | | |
| 16 | ● | | 65 | 76 | 55 | 30.00 | 29.95 | 63 | 65 | ENE | ESE | | | |
| 17 | | | 62 | 68 | 49 | 30.00 | 30.00 | 75 | 65 | ENE | NE | Cloud. | Cloud. | |
| 18 | | | 56 | 68 | 56 | 30.00 | 29.94 | 65 | 68 | E | SE | Fine | Fine | |
| 19 | | | 61 | 71 | 55 | 29.92 | 29.65 | 63 | 58 | W | NW | Cloud. | | |

The quantity of rain fallen in the month of May, was 1 inch and 75.100ths.

